# Regulating Protein Corona Formation and Dynamic Protein Exchange by Controlling Nanoparticle Hydrophobicity

**DOI:** 10.3389/fbioe.2020.00210

**Published:** 2020-03-20

**Authors:** Qianhui Yu, Linxia Zhao, Congcong Guo, Bing Yan, Gaoxing Su

**Affiliations:** ^1^School of Environmental Science and Engineering, Shandong University, Qingdao, China; ^2^School of Pharmacy, Nantong University, Nantong, China; ^3^Key Laboratory for Water Quality and Conservation of the Pearl River Delta, Institute of Environmental Research at Greater Bay, Ministry of Education, Guangzhou University, Guangzhou, China

**Keywords:** surface chemistry, hydrophobicity, protein corona, nanoparticles, nano-bio interactions

## Abstract

Physiochemical properties of engineered nanoparticles (NPs) play a vital role in nano-bio interactions, which are critical for nanotoxicity and nanomedicine research. To understand the effects of NP hydrophobicity on the formation of the protein corona, we synthesized four gold NPs with a continuous change in hydrophobicity ranging from −2.6 to 2.4. Hydrophobic NPs adsorbed 2.1-fold proteins compared to hydrophilic ones. Proteins with small molecular weights (<50 kDa) and negatively charge (PI < 7) constituted the majority of the protein corona, especially for hydrophobic NPs. Moreover, proteins preferred binding to hydrophilic NPs (vitronectin and antithrombin III), hydrophobic NPs (serum albumin and hemoglobin fetal subunit beta), and medium hydrophobic NPs (talin 1 and prothrombin) were identified. Besides, proteins such as apolipoprotein bound to all NPs, did not show surface preference. We also found that there was a dynamic exchange between hard protein corona and solution proteins. Because of such dynamic exchanges, protein-bound NPs could expose their surface in biological systems. Hydrophilic NPs exhibited higher protein exchange rate than hydrophobic NPs. Above understandings have improved our capabilities to modulate protein corona formation by controlling surface chemistry of NPs. These will also help modulate nanotoxicity and develop better nanomedcines.

## Introduction

Engineered nanoparticles (NPs) with unique physical and chemical properties have been widely used in catalysis (Liu and Dai, 2016; Sharma et al., 2015), electronics (Liu et al., 2017; Wu, 2017), and biomedicine (Ramos et al., 2017; Ke et al., 2018). Until now, more than 3 000 nanomaterial-based consumer products are on the market (Wei and Yan, 2016). These applications will increase the risk of human exposure to engineered NPs. To understand possible health issues of engineered NPs, it is necessary to clarify the basic interactions between NPs and physiological systems, blood, and biomolecules (Nel et al., 2009; Srivastava et al., 2015). Such understandings will significantly facilitate design of nanomedicine with well-defined pharmacokinetics and biodistribution (Walkey et al., 2012; Su et al., 2018). Therefore, understanding and tailoring the fundamental interactions between NPs and physiological systems has become a focus of nanotoxicity and nanomedicine research.

Physiological environments, such as blood, interstitial fluid, and cellular cytoplasm, contain complex protein mixtures. When engineered NPs enter such physiological environment, they spontaneously adsorb proteins to form protein corona (Cedervall et al., 2007a, b; Lundqvist et al., 2008; Ke et al., 2017). Protein corona may consist of tens or hundreds of proteins. They alter the physicochemical properties of NPs, such as size, zeta potential, morphology, and aggregation state (Gebauer et al., 2012; Su et al., 2012; Glancy et al., 2019; Marichal et al., 2019). At the same time, the protein corona also alters the interactions between NPs and biological systems and modulates the kinetics, transport, and reactivity of NPs (Monopoli et al., 2011; Lesniak et al., 2012; Walkey et al., 2012, 2014; Tenzer et al., 2013). For example, adsorbed proteins may act as opsonins, and dramatically enhanced the uptake of NPs by phagocytes (Walkey et al., 2012). Recent studies have shown that the synthetic identity of NPs plays an important role in determining the composition of the protein corona and the subsequent cellular interactions (Walkey et al., 2014; Caracciolo et al., 2015). Effects of size, shape, and surface chemistry of a NP on the protein corona formation were also studied (Johnston et al., 2017; Nienhaus and Nienhaus, 2019; Walkey et al., 2012; Su et al., 2016). Smaller NPs adsorb relatively more proteins compared to larger NPs due to a larger surface area in smaller NPs. Porous particles decreased the deposition of adsorbed proteins due to the size-exclusion effect (Clemments et al., 2015). Coating NPs with polyethylene glycol (PEG) or polysaccharides can minimize the protein adsorption (Walkey et al., 2012; Schöttler et al., 2016; Yahyaei et al., 2018). As the coating density increasing, less proteins are adsorbed. Although efforts have been made to minimize protein adsorption on NPs, systematic understanding of the relationships between the well-controlled NP’s surface physiochemical properties and protein corona formation is relatively few.

The hydrophobic interaction is one of the most important interactions between molecules. It may be also so between NPs and proteins (Mahmoudi et al., 2011; Shemetov et al., 2012; Zhong et al., 2014). Foreign hydrophobic molecules or NPs are harmful to biological systems by disrupting cell membrane and protein folding. On the other hand, a certain degree of hydrophobicity was needed for drugs to cross cell membranes or biological barriers (Cunningham et al., 2018). Protein corona formation may change the hydrophobicity of NPs. Meanwhile, hydrophobicity of NPs may determine the nature of protein corona (Ashby et al., 2014; Vasti et al., 2016; Pareek et al., 2018). However, most reports were limited to NPs with a narrow distribution of LogP range or surface ligands with different molecular structures making comparison difficult. In most cases, LogP values of NPs were not carefully characterized. In this work, we assembled NPs with a continuous change in surface hydrophobicity with identical size, shape and core material to investigate protein corona formation on these NPs. LogP values of these NPs were ranging from −2.6 to + 2.4, as measured by shaking-flask method. Due to the hydrophobic interactions, hydrophobic NPs adsorbed more than twice proteins of hydrophilic NPs. Proteomics analysis of protein corona was also carried out by nano-LC-MS/MS to identify proteins on NPs. Small and negatively charged proteins constituted the majority of adsorbed proteins. Moreover, adsorbed proteins were loosely associated to NPs and were dynamically exchanging with proteins in solution. The original physicochemical properties of NPs were mostly maintained in physiological environment. This study helps us understand protein corona formation in order to regulate corona in various applications.

## Experimental Section

### Materials

Ligand A and B were prepared as we previously reported (Li et al., 2015). Other chemicals were purchased from Sigma-Aldrich without purification. Glassware used in this study were immersed in aqua regia overnight and then washed with ultrapure water several times.

### Synthesis of Modified Gold NPs

Hydrogen tetrachloroaurate (III) trihydrate (HAuCl_4_ ⋅ 3H_2_O, 0.032 mmol) was added into H_2_O (0.625 mL). A solution of mixture of ligand A and ligand B in different ratios in DMF (0.625 mL) was added to the mixture. After stirring for 30 min, ice-cold NaBH_4_ solution (0.131 mmol, 0.5 mL) was added to the mixture. The mixture solution turned red immediately and was vigorously stirred for 1 h. After washing DMF and water five times, as-prepared NPs were dispersed in 5 mL of water and kept at 4°C until use. The ratios of ligand A and ligand B on gold NPs were measured by detaching the ligands using I_2_ and performing high-performance liquid chromatograph (Li et al., 2015). If the ratios were not the same as our design, ratios of ligand A and ligand B were adjusted and measured once again.

### Characterization

The morphologies of gold NPs were characterized by transmission electron microscopy (TEM) (JEM-1011, JEOL, operating at 100 kV). Hydrodynamic diameters and zeta potentials were characterized by dynamic light scattering (NanoBrook 90Plus Zeta, Brookheaven). Before measuring, these NPs should be sonicated several minutes to help disperse. The concentrations of each NP’s stock solution were detected by inductively coupled plasma mass spectrometry (ICP-MS, Agilent).

### LogP Measurements

First, octanol and water were mixed for 24 h, and octanol-saturated water and water-saturated octanol were obtained. NPs (0.1 mg) were diluted into octanol-saturated water (2 mL), and water-saturated octanol (2 mL) were added. The mixture was shaken for 24 h at room temperature and then stood still for 3 h. NPs were separation from the two phases. After digesting with aqua regia, concentrations of NPs were measured by ICP-MS. The LogP values can be calculated according: LogP = LogC (NP in Octanol)/LogC (NP in Water).

### Protein Adsorption

After sonicating for several minutes, NPs (0.1 mg) were added to PBS (1 mL) containing 10% fetal bovine serum (FBS). The mixture was kept at 37°C water bath for 1 h. Then, the mixture was centrifuged, the pellet was washed with PBS three times and NPs with protein corona were obtained.

### SDS-PAGE

Nanoparticles with protein corona were dispersed into PBS. LDS loading buffer and 2-Mercaptoethanol were added and heating at boiling temperature for 5 min to release the bound proteins. After centrifugation, the supernatant was collected to run the sodium dodecyl sulfate-poly-(acrylamide gel electrophoresis) (SDS-PAGE). At last, the gels were washed with water several times and stained with coomassie brilliant blue following the protocol.

### BCA Assays

According to previous report (Su et al., 2018), the protein corona were recovered by sonicating the protein-bound NPs in extraction buffer (Tris–HCl buffer, pH 6.8, 10% glycerol, and 4% SDS) for several minutes. After centrifugation, the supernatant was collected and the protein concentration was measured by the BCA assay kit (Beyotime Biotechnology) according to manufacturer’s protocol.

### Nano-LC-MS/MS

For in solution digestion, a protein solution sample was first reduced by DTT and all Cysteine residues alkylated by iodoacetamide and cleaned by desalting columns or ethanol precipitation. The sample was then digested with sequencing grade modified trypsin (Promega) in the digestion buffer (ammonium bicarbonate 100 mM, pH 8.5). A dissolved peptide sample is then analyzed by a Nano-LC-ESI-MS/MS system.

Nano-LC-ESI-MS/MS analysis of a digested protein sample was carried out by a high-pressure liquid chromatography (HPLC) system (Agilent) with a 75 um ID 8 cm in length in house packed reverse phase C18 capillary column. The particle size of the C18 column is 3 μM and the pore size is 300 Å. The sample injection time was 20 min. The HPLC Solvent A was 97.5% water, 2% acetonitrile, 0.5% formic acid. HPLC Solvent B is 9.5% water, 90% acetonitrile, and 0.5% formic acid. The gradation time was 60 min from 2% Solvent B to 90% solvent B, plus 20 min for sample loading, and 20 min for column washing. The column flow rate was around 800 nL per min after splitting. Typical sample injection volume is 3 μL.

### Protein Exchange Experiments

Firstly, NPs (0.5 mg) were mixed with 0.1 mg/mL FITC-labeled BSA solution. After shaking at 37°C for 1 h to form protein corona, the mixture was centrifuged at 20000g (4°C, 1 h) and washed with PBS twice. Then, the supernatants were combined and the fluorescence intensity was measured. According to a calibration curve, amounts of unadsorbed FITC-labeled BSA was calculated. The total amount of adsorbed FITC-labeled BSA was calculated by subtracting the amount of unadsorbed FITC-labeled BSA from the total amount of FITC-labeled BSA.

The NPs with fluorescence corona were then redispersed into 0.1 mg/mL non-labeled BSA solution. At different time points, a fraction of the solutions was centrifuged and the fluorescence intensity of the supernatant was determined. According to a calibration curve, amounts of exchanged FITC-labeled BSA were determined. The exchange rate was calculated by dividing the amount of exchanged FITC-labeled BSA with the amount of adsorbed FITC-labeled BSA.

## Results and Discussion

### Synthesis and Characterization of Nanoparticles With a Continuous Change in Hydrophobicity

**FIGURE 1:**
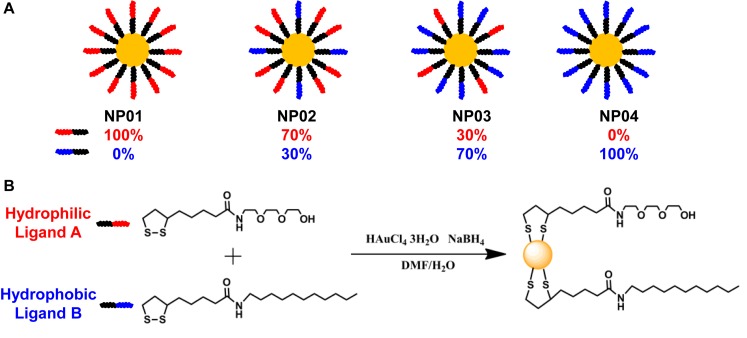
**(A)** Four nanoparticles (NP01∼NP04) were synthesized with continuous change in hydrophobicity. **(B)** Ligand chemical structures and the synthesis route.

**FIGURE 2:**
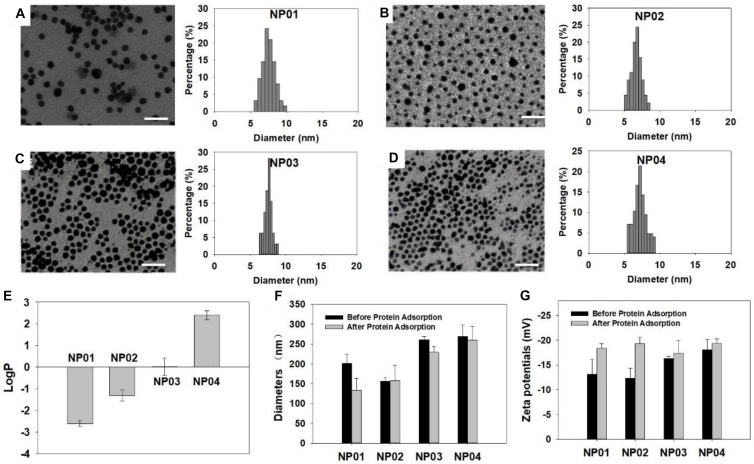
Characterization of NPs. **(A–D)** TEM images and size distribution of NPs. **(A)** NP01, **(B)** NP02, **(C)** NP03, **(D)** NP04. Scale bar: 25 nm. **(E)** LogP values of NPs. **(F)** Hydrodynamic diameters and **(G)** zeta potentials of NPs before and after protein adsorption.

A NP array (NP01∼NP04) with a continuous change in hydrophobicity was synthesized and their chemical structures were shown in Figure 1A. Two ligands were used: hydrophilic ligand A with a tri-ethylene glycol, and hydrophobic ligand B with an undecane (Figure 1B). During the reduction of gold, ligands were attached *in situ* to gold NP surface through formation of Au-S bond. By redundantly adjusting the ratios of ligands A and B in the reaction solution, NPs coated with various ratios of ligand A and B (such as 30% or 70%) were obtained. The advantage of this strategy is that NP products were only different in hydrophobicity, while their size, shape, and core materials were controlled identical. The average core diameters of gold NPs were 7.6 ± 0.9 nm (NP01), 6.7 ± 1.0 nm (NP02), 7.5 ± 0.9 nm (NP03), and 7.1 ± 1.1 nm (NP04) as characterized by transmission electrical microscopy (TEM) (Figures 2A–D).

We have previously shown that LogP value of surface ligand did not predict LogP of NPs (Li et al., 2015) and therefore, we experimentally determined the LogP values of these NPs using “shaking flask” method. Their LogP values were ranging from −2.6 to 2.4 (Figure 2E). This range is wide enough to represent most NPs used in various applications nowadays. In aqueous solution, hydrodynamic diameters of the NP array were in a range of 150∼300 nm (Figure 2F). Protein adsorption will help NP suspend. We observed that the hydrodynamic diameters of several NPs decreased after protein adsorption. All NPs exhibited negatively charged surface in water with zeta potential values around −20 mV (Figure 2G). After protein adsorption, zeta potentials did not change much. These results revealed that protein adsorption could influence the physicochemical properties of NPs in some way.

### Nanoparticles With Higher Hydrophobic Surface Adsorbed More Proteins

**FIGURE 3:**
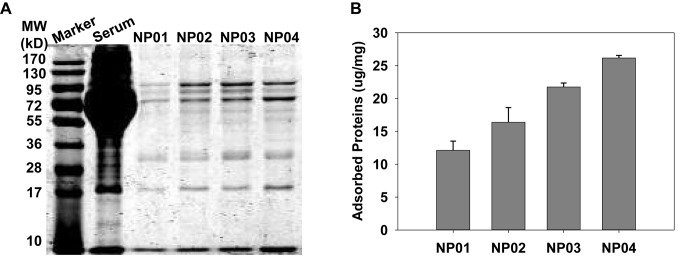
**(A)** Qualitative characterization of protein corona on nanoparticles using SDS-PAGE. **(B)** Quantitative analysis of the amounts of adsorbed proteins by BCA assays.

To understand the impacts of NP hydrophobicity on the formation of protein corona, we quantitatively and qualitatively analyzed of adsorbed proteins by NPs. First, the protein corona was analyzed using SDS-PAGE after proteins were dissociated from NPs (Figure 3A). After Coomassie brilliant blue staining, many protein bands appeared, indicating that various serum proteins were adsorbed to NP surface. The molecular weight of each band represented protein identity, while the intensity of each band reflected amounts of adsorbed proteins. We observed that band intensity gradual increased with the NP hydrophobicity increasing, suggesting more proteins were bound to NPs with higher hydrophobicity. In a more quantitative measurement, the isolated proteins were quantitatively analyzed by BCA assays (Figure 3B). More hydrophilic NP01 adsorbed about 12 μg proteins per milligram of NPs while the amounts of adsorbed proteins increased with NP hydrophobicity to about 26 μg proteins per milligram of NPs. This trend was consistent with the results of SDS-PAGE. Hydrophobic NPs with higher surface energy (Mandal et al., 2012) provides stronger hydrophobic interactions between NPs and proteins, resulted in increased protein adsorption.

### Small and Negatively Charged Proteins Were Preferably Adsorbed to NPs With Hydrophobic Surface

The composition analysis of the protein corona on four NPs was analyzed using electrospray nano-liquid chromatography mass spectrometry (nano-LC-MS/MS) (Griffin et al., 2010; Eeltink et al., 2017). Identified proteins were listed in Supplementary Table S1. Total 21, 58, 82, and 41 proteins in detectable quantity were found on NP01, NP02, NP03, and NP04, respectively.

**FIGURE 4:**
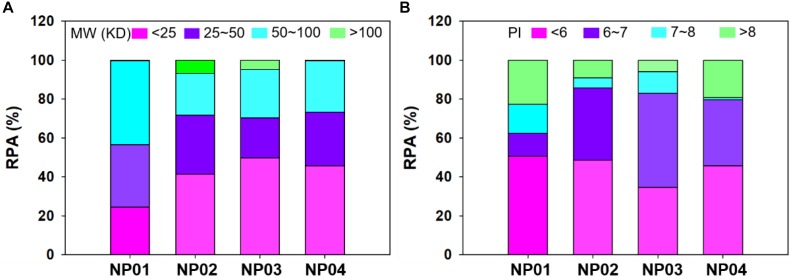
Relative protein abundance of protein corona classified according to their molecular mass **(A)** and isoelectric point **(B)**.

Relative abundance (RPA) of corona proteins was also determined by nano-LC-MS/MS. We first classified proteins by their molecular weights and isoelectric points. As shown in Figure 4A, NPs with different hydrophobicity were able to selectively bind different proteins according to their molecular weights. Due to large surface curvature of 7 nm NPs, all NPs exhibited low affinity for proteins >100 kDa, while proteins <100 kDa accounted for more than 90% of the protein corona. In particular, NP02, NP03 and NP04 exhibited strong affinity for proteins with molecular weights < 50 kDa (about 71, 70, and 73% for NP02, NP03 and NP04, respectively), while NP01 even though adsorbed maily proteins between <100 KDa (99%), it adsorbed twice as much proteins with molecular weights between 50 and 100 KDa (about 43%) compared to other three NPs (about 21, 24, and 26%). Finally, the amount of proteins <25 kDa in the corona of NP01 was a half (about 24%) compared to other three NPs (41∼49%). The results indicating the distribution of protein molecular weight was similar between medium hydrophobic NPs and high hydrophobic NPs, but different with hydrophilic NPs.

Further analyses were performed to understand the relationship between protein isoelectric point and protein corona. Figure 4B shows that the largest fraction of corona proteins has a negative charge (isoelectric point, pI < 7) (about 61, 85, 82, and 79% for NP01, NP02, NP03, and NP04, respectively). Moreover, NP01, NP02, and NP04 adsorbed mainly proteins with a pI < 6 (about 50% for the three NPs), while NP03 adsorbed mainly proteins with a pI between 6 and 7 (48%). NP01 and NP04 adsorbed twofold proteins with a pI > 8 (about 20%) compared to other two NPs. NP01 adsorbed lowest abundance of proteins with PI 6∼7 and highest abundance of proteins with PI 7∼8 compared to other three NPs. PIs represent the electronic distribution of adsorbed proteins and possible electrostatic interactions between NPs and proteins. Although the conjugated ligands were neutral and zeta potentials of NPs were medium negative, proteins with negative charge in solution were more preferred binding to NPs. The stronger bindings between these proteins and NPs were possible mainly through the hydrogen bonds and hydrophobic forces for hydrophilic NPs and hydrophobic NPs, respectively.

### Identification of Proteins Binding to NPs With Different Hydrophobicity

**FIGURE 5:**
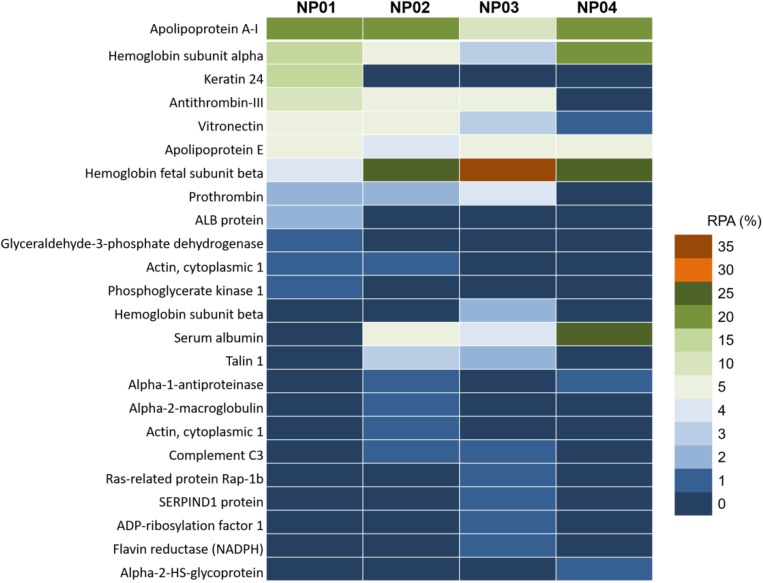
Nano-LC-MS/MS label-free proteomic analysis heat map of the abundant proteins (>1%) of protein corona on NP01, NP02, NP03, and NP04.

Protein composition analysis showed that top 10 bound proteins constituted about 80–90% of the total adsorbed proteins (Figure 5 and Supplementary Table S1). Particularly, for NP04, top 10 and top 5 bound proteins constituted 96% and 91% of the total protein content, respectively. The results indicated that relatively few types of proteins were enriched by NPs from thousands of serum proteins. Apolipoprotein A-I and Apolipoprotein E were adsorbed to all NPs with similar RPA. The hydrophobic forces may be not involved in the interactions between NPs and these proteins. As surface hydrophobic increasing, NPs adsorbed more hemoglobin fetal subunit beta (from 4% to 35%) and serum albumin (from 0.3% to 23%), indicating these proteins would prefer binding to hydrophobic surface with involvement of strong hydrophobic forces. On the other hand, the relative amounts of some proteins in the corona decreased as the surface hydrophobicity increased. Such proteins included vitronectin (decreased from 6.7% to 1.1%), and antithrombin III (decreased from 14.3% to 0.5%). Besides, talin 1 and prothrombin were found to prefer binding to surface with medium hydrophobicity. Interactions between NPs and proteins are complicated. Fully understanding the mechanisms of interactions between these proteins and NP surface with different hydrophobicity should consider the three-dimension structure of these proteins (Khan et al., 2013; Kharazian et al., 2016). In our further work, we will use computation modeling to investigate the related mechanisms.

Consistent with previous reported results, the amounts of the proteins in the corona were not correlated with their relative abundance in the serum (Sakulkhu et al., 2015; Vidaurre-Agut et al., 2019). For example, serum digestion and analysis confirmed that serum albumin (about 60% in serum) was one of the most abundant proteins found in the 10% FBS used for these experiments, but it was found in relatively low abundance on NP01, NP02, and NP03, constituting less than 5% of the complete corona. Similarly, one of the most abundant serum proteins, serotransferrin (about 4% in serum), was only identified on NP03 with the RPA of 0.1%. On the other hand, hemoglobin subunit alpha and hemoglobin fetal subunit beta found in trace concentrations in serum, however, was the major component of the corona of all particles. Therefore, NPs with different surface chemistry can be used to enrich certain proteins for proteomic research (Wang et al., 2018; Yao et al., 2018).

### Protein Exchange Rate Was Higher on Hydrophilic NPs Than Hydrophobic NPs

**FIGURE 6:**
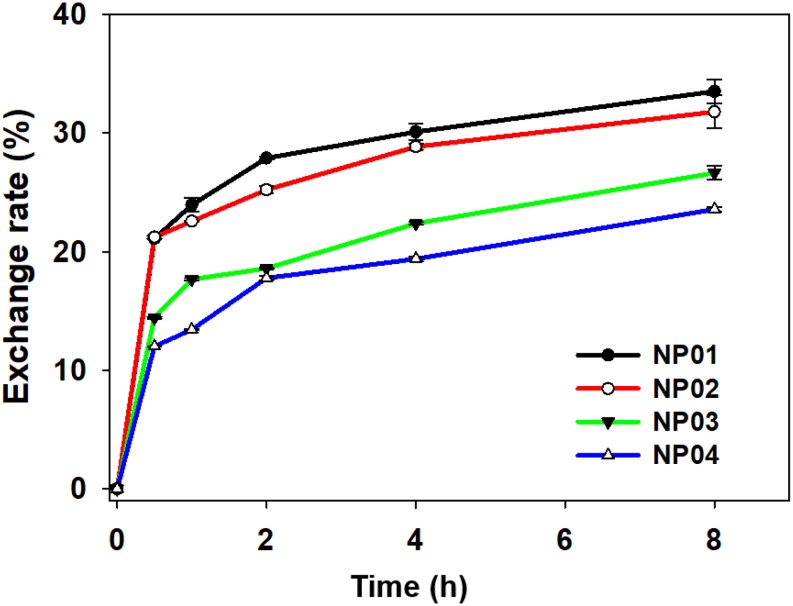
Dynamic protein exchange between FITC-labeled BSA and non-labeled BSA.

Protein corona has shown notable impact on the biological behavior of NPs in biological systems. However, a puzzling dilemma is whether hard protein corona completely shields a NP or the NP is still exposed? To test this, we designed an experiment to examine protein exchange between NPs covered with fluorescence-labeled protein corona and non-fluorescence-labeled proteins in solution. A labeled model protein, FITC-labeled bovine fetal albumin (BSA), was used to form hard protein corona with NP01-NP04 in this study. When FITC-labeled BSA was adsorbed to NPs, the fluorescence of FITC was partially quenched by gold NPs. Protein-covered NPs were isolated by centrifugation and washed with PBS, leaving only hard protein corona on NP01-NP04. After exchanging with non-labeled proteins in the solution, the fluorescence of FITC in the supernatant was continuously measured. The protein exchange rates were calculated based on the fluorescence intensity. As shown in Figure 6, after 8 h incubation, the highest protein exchange rate happened on the surface of NP01, reached 34%, while the lowest took place on NP04, was 23%. The experimental findings demonstrated that hard corona was not binding as tightly as covalent bindings. These non-covalently bound proteins were freely exchange with counterparts in solution. Therefore, NPs are not completely shielded. Hydrophobic surface adsorbed about twofold more proteins than hydrophilic surface (see the results of BCA assays), and the dense packed adsorbed proteins restricted the exchange with free proteins in the solution.

Numerous studies have reported nano-bio interactions were correlated to NPs’ original physicochemical properties, including hydrophobicity, charge, ligand structure, and core composition. For example, NPs modified with targeting moieties showed enhanced cellular internalization in protein-rich medium. The dynamic exchange behavior of adsorbed proteins told us NPs could expose themselves in physiological systems even though proteins were coated at the outmost layer. Adsorbed proteins were loosely associated with NPs and they also underwent quick and frequent exchanges with proteins in solution. Therefore, protein corona does not block the original physicochemical properties of NPs.

## Conclusion

To study the relationships between surface hydrophobicity and the formation and dynamic behavior of the protein corona, a NP array was synthesized with a wide range of surface hydrophobicity with LogP values ranging from −2.6 to +2.4. Hydrophobic NP surface adsorbed 2.1-fold proteins compared to hydrophilic ones which was attributed to the stronger hydrophobic interactions. Due to large surface curvature and electrostatic interactions, the most adsorbed proteins had small molecular weights and negatively charge, especially for hydrophobic surface. Apolipoproteins were adsorbed to all types of NPs, with no significant differences on bound amount. On the other hand, hemoglobin fetal subunit beta and serum albumin preferred binding to hydrophobic NPs, while vitronectin and antithrombin III preferred binding to hydrophilic NPs. Furthermore, hydrophilic NPs exhibited a higher hard corona protein exchange rate than the hydrophobic NPs. These findings enhanced our understanding on the control of protein adsorption and protein exchange dynamics. These understandings will help advance the design of the next generation of nanomedicines.

## Data Availability Statement

The data found in this study can be found on ProteomeXchange, accession number PXD017429.

## Author Contributions

BY and GS conceived this project, designed the methodology, and participated in writing the manuscript. QY synthesized the nanoparticles. QY, LZ, and CG performed the rest of the experiments. All authors have read and approved the final manuscript.

## Conflict of Interest

The authors declare that the research was conducted in the absence of any commercial or financial relationships that could be construed as a potential conflict of interest.
