# Supplementary material for: Regulating Protein Corona Formation and Dynamic Protein Exchange by Controlling Nanoparticle Hydrophobicity
Source: Front Bioeng Biotechnol. 2020 Mar 20;8:210. doi: 10.3389/fbioe.2020.00210 (PMC7100549; doi:10.3389/fbioe.2020.00210)
Supplement: Supplementary file 1 [file Data_Sheet_1.pdf]

Supporting Information for

Regulating Protein Corona Formation and Dynamic Protein Exchange by Controlling Nanoparticle Hydrophobicity

Qianhui Yu,[1] Linxia Zhao,[2] Congcong Guo,[1] Bing Yan,[*,3,1] Gaoxing Su[*,2]

[1]School of Environmental Science and Engineering, Shandong University, Qingdao, China

[2]School of Pharmacy, Nantong University, Nantong, China

[3]Institute of Environmental Research at Greater Bay, Key Laboratory for Water Quality and Conservation of the Pearl River Delta, Ministry of Education, Guangzhou University, Guangzhou, China

[*]Corresponding Authors:

Bing Yan: E-mail: drbingyan@yahoo.com

Gaoxing Su: E-mail: sugaoxing@ntu.edu.cn

Table S1. List of proteins with relative abundance identified by nano-LC-MS/MS.

	Identified Proteins	Accession Number	Molecular Weight	RPA
**NP01**				
1	>tr|V6F9A2|V6F9A2_BOVIN Apolipoprotein A-I preproprotein OS=Bos taurus GN=APOA1 PE=3 SV=1	V6F9A2	30257.9	20.05%
2	>sp|P01966|HBA_BOVIN Hemoglobin subunit alpha OS=Bos taurus GN=HBA PE=1 SV=2	P01966	15174.93	19.33%
3	>tr|F1MFW9|F1MFW9_BOVIN Keratin 24 OS=Bos taurus GN=KRT24 PE=3 SV=2	F1MFW9	50903.66	16.45%
4	>sp|P41361|ANT3_BOVIN Antithrombin-III OS=Bos taurus GN=SERPINC1 PE=1 SV=2	P41361	52827.16	14.32%
5	>tr|Q3ZBS7|Q3ZBS7_BOVIN Vitronectin OS=Bos taurus GN=VTN PE=1 SV=1	Q3ZBS7	54339.32	6.75%
6	>tr|A0A140T881|A0A140T881_BOVIN Apolipoprotein E OS=Bos taurus GN=APOE PE=3 SV=1	A0A140T881	36073.61	6.54%
7	>sp|P02081|HBBF_BOVIN Hemoglobin fetal subunit beta OS=Bos taurus PE=1 SV=1	P02081	15963.34	4.28%
8	>sp|P00735|THRB_BOVIN Prothrombin OS=Bos taurus GN=F2 PE=1 SV=2	P00735	71886.34	2.72%
9	>tr|B0JYQ0|B0JYQ0_BOVIN ALB protein OS=Bos taurus GN=ALB PE=2 SV=1	B0JYQ0	71244.36	2.34%
10	>sp|P10096|G3P_BOVIN Glyceraldehyde-3-phosphate dehydrogenase OS=Bos taurus GN=GAPDH PE=1 SV=4	P10096	36073.38	1.66%
11	>sp|P60712|ACTB_BOVIN Actin, cytoplasmic 1 OS=Bos taurus GN=ACTB PE=1 SV=1	P60712	42051.85	1.50%
12	>sp|Q3T0P6|PGK1_BOVIN Phosphoglycerate kinase 1 OS=Bos taurus GN=PGK1 PE=2 SV=3	Q3T0P6	44908.24	1.08%
13	>sp|P34955|A1AT_BOVIN Alpha-1-antiproteinase OS=Bos taurus GN=SERPINA1 PE=1 SV=1	P34955	46417.03	0.89%
14	>sp|P02070|HBB_BOVIN Hemoglobin subunit beta OS=Bos taurus GN=HBB PE=1 SV=1	P02070	16001.32	0.59%
15	>tr|G5E5T5|G5E5T5_BOVIN Uncharacterized protein OS=Bos taurus PE=1 SV=1	G5E5T5	44457.32	0.41%
16	>sp|F1MF74|MICA2_BOVIN [F-actin]-methionine sulfoxide oxidase MICAL2 OS=Bos taurus GN=MICAL2 PE=3 SV=2	F1MF74	125132.88	0.40%
17	>sp|P02769|ALBU_BOVIN Serum albumin OS=Bos taurus GN=ALB PE=1 SV=4	P02769	71244.31	0.36%
18	>tr|F6RQK3|F6RQK3_BOVIN Uncharacterized protein	F6RQK3	24417.63	0.17%

<table>
<tr><td></td><td>OS=Bos taurus GN=GSTZ1 PE=1 SV=1</td><td></td><td></td><td></td></tr>
<tr><td>19</td><td>>sp|Q5E9B1|LDHB_BOVIN L-lactate dehydrogenase B chain OS=Bos taurus GN=LDHB PE=2 SV=4</td><td>Q5E9B1</td><td>36985.35</td><td>0.11%</td></tr>
<tr><td>20</td><td>>sp|Q28065|C4BPA_BOVIN C4b-binding protein alpha chain OS=Bos taurus GN=C4BPA PE=2 SV=1</td><td>Q28065</td><td>70951.3</td><td>0.03%</td></tr>
<tr><td>21</td><td>>tr|A5D984|A5D984_BOVIN Pyruvate kinase OS=Bos taurus GN=PKM2 PE=1 SV=1</td><td>A5D984</td><td>58482.34</td><td>0.03%</td></tr>
<tr><td colspan="5">NP02</td></tr>
<tr><td>1</td><td>>sp|P02081|HBBF_BOVIN Hemoglobin fetal subunit beta OS=Bos taurus PE=1 SV=1</td><td>P02081</td><td>15963.34</td><td>29.73%</td></tr>
<tr><td>2</td><td>>tr|V6F9A2|V6F9A2_BOVIN Apolipoprotein A-I preproprotein OS=Bos taurus GN=APOA1 PE=3 SV=1</td><td>V6F9A2</td><td>30257.9</td><td>21.94%</td></tr>
<tr><td>3</td><td>>sp|P01966|HBA_BOVIN Hemoglobin subunit alpha OS=Bos taurus GN=HBA PE=1 SV=2</td><td>P01966</td><td>15174.93</td><td>7.27%</td></tr>
<tr><td>4</td><td>>tr|Q3ZBS7|Q3ZBS7_BOVIN Vitronectin OS=Bos taurus GN=VTN PE=1 SV=1</td><td>Q3ZBS7</td><td>54339.32</td><td>6.36%</td></tr>
<tr><td>5</td><td>>sp|P02769|ALBU_BOVIN Serum albumin OS=Bos taurus GN=ALB PE=1 SV=4</td><td>P02769</td><td>71244.31</td><td>5.90%</td></tr>
<tr><td>6</td><td>>sp|P41361|ANT3_BOVIN Antithrombin-III OS=Bos taurus GN=SERPINC1 PE=1 SV=2</td><td>P41361</td><td>52827.16</td><td>5.09%</td></tr>
<tr><td>7</td><td>>tr|A0A140T881|A0A140T881_BOVIN Apolipoprotein E OS=Bos taurus GN=APOE PE=3 SV=1</td><td>A0A140T881</td><td>36073.61</td><td>4.27%</td></tr>
<tr><td>8</td><td>>tr|G3N0S9|G3N0S9_BOVIN Uncharacterized protein OS=Bos taurus GN=LOC515150 PE=4 SV=1</td><td>G3N0S9</td><td>22948.58</td><td>3.60%</td></tr>
<tr><td>9</td><td>>tr|F1MDH3|F1MDH3_BOVIN Talin 1 OS=Bos taurus GN=TLN1 PE=1 SV=2</td><td>F1MDH3</td><td>272810.53</td><td>3.47%</td></tr>
<tr><td>10</td><td>>sp|P00735|THRB_BOVIN Prothrombin OS=Bos taurus GN=F2 PE=1 SV=2</td><td>P00735</td><td>71886.34</td><td>2.37%</td></tr>
<tr><td>11</td><td>>sp|P34955|A1AT_BOVIN Alpha-1-antiproteinase OS=Bos taurus GN=SERPINA1 PE=1 SV=1</td><td>P34955</td><td>46417.03</td><td>1.36%</td></tr>
<tr><td>12</td><td>>sp|Q7SIH1|A2MG_BOVIN Alpha-2-macroglobulin OS=Bos taurus GN=A2M PE=1 SV=2</td><td>Q7SIH1</td><td>168952.25</td><td>1.29%</td></tr>
<tr><td>13</td><td>>sp|P60712|ACTB_BOVIN Actin, cytoplasmic 1 OS=Bos taurus GN=ACTB PE=1 SV=1</td><td>P60712</td><td>42051.85</td><td>1.02%</td></tr>
<tr><td>14</td><td>>sp|Q2UVX4|CO3_BOVIN Complement C3 OS=Bos taurus GN=C3 PE=1 SV=2</td><td>Q2UVX4</td><td>188674</td><td>1.01%</td></tr>
<tr><td>15</td><td>>sp|P10096|G3P_BOVIN Glyceraldehyde-3-phosphate dehydrogenase OS=Bos taurus GN=GAPDH PE=1 SV=4</td><td>P10096</td><td>36073.38</td><td>0.96%</td></tr>
<tr><td>16</td><td>>sp|Q28178|TSP1_BOVIN Thrombospondin-1 OS=Bos taurus GN=THBS1 PE=2 SV=2</td><td>Q28178</td><td>133441.97</td><td>0.76%</td></tr>
<tr><td>17</td><td>>tr|F1MFW9|F1MFW9_BOVIN Keratin 24 OS=Bos taurus GN=KRT24 PE=3 SV=2</td><td>F1MFW9</td><td>50903.66</td><td>0.65%</td></tr>
</table>

18	>tr|A6QPP2|A6QPP2_BOVIN SERPIND1 protein OS=Bos taurus GN=SERPIND1 PE=2 SV=1	A6QPP2	55457.54	0.40%
19	>tr|Q3ZBQ9|Q3ZBQ9_BOVIN APOM protein OS=Bos taurus GN=APOM PE=2 SV=1	Q3ZBQ9	13303.59	0.40%
20	>sp|P17697|CLUS_BOVIN Clusterin OS=Bos taurus GN=CLU PE=1 SV=1	P17697	51651.37	0.27%
21	>tr|F1MZC0|F1MZC0_BOVIN Uncharacterized protein OS=Bos taurus PE=1 SV=1	F1MZC0	30425.02	0.21%
22	>sp|Q9XSJ4|ENOA_BOVIN Alpha-enolase OS=Bos taurus GN=ENO1 PE=1 SV=4	Q9XSJ4	47638.55	0.20%
23	>sp|P61223|RAP1B_BOVIN Ras-related protein Rap-1b OS=Bos taurus GN=RAP1B PE=2 SV=1	P61223	21039.67	0.18%
24	>sp|Q28107|FA5_BOVIN Coagulation factor V OS=Bos taurus GN=F5 PE=1 SV=1	Q28107	249909.67	0.13%
25	>sp|Q3ZCJ7|TBA1C_BOVIN Tubulin alpha-1C chain OS=Bos taurus GN=TUBA1C PE=1 SV=1	Q3ZCJ7	50509.62	0.11%
26	>sp|P52556|BLVRB_BOVIN Flavin reductase (NADPH) OS=Bos taurus GN=BLVRB PE=1 SV=2	P52556	22232.4	0.10%
27	>tr|G5E513|G5E513_BOVIN Uncharacterized protein OS=Bos taurus PE=1 SV=1	G5E513	50622.92	0.08%
28	>sp|P55918|MFAP4_BOVIN Microfibril-associated glycoprotein 4 OS=Bos taurus GN=MFAP4 PE=1 SV=2	P55918	28882.01	0.08%
29	>tr|E1BHF6|E1BHF6_BOVIN Uncharacterized protein OS=Bos taurus GN=SIPA1L3 PE=4 SV=2	E1BHF6	198294.25	0.08%
30	>sp|Q3SZV7|HEMO_BOVIN Hemopexin OS=Bos taurus GN=HPX PE=2 SV=1	Q3SZV7	52974.13	0.07%
31	>sp|P12763|FETUA_BOVIN Alpha-2-HS-glycoprotein OS=Bos taurus GN=AHSG PE=1 SV=2	P12763	39192.69	0.07%
32	>sp|Q3SYV4|CAP1_BOVIN Adenylyl cyclase-associated protein 1 OS=Bos taurus GN=CAP1 PE=2 SV=3	Q3SYV4	51582.59	0.06%
33	>sp|Q9TTJ5|RGN_BOVIN Regucalcin OS=Bos taurus GN=RGN PE=2 SV=1	Q9TTJ5	33856.69	0.05%
34	>tr|A5D984|A5D984_BOVIN Pyruvate kinase OS=Bos taurus GN=PKM2 PE=1 SV=1	A5D984	58482.34	0.05%
35	>sp|Q3MHN5|VTDB_BOVIN Vitamin D-binding protein OS=Bos taurus GN=GC PE=2 SV=1	Q3MHN5	54903.71	0.04%
36	>sp|Q5E9F5|TAGL2_BOVIN Transgelin-2 OS=Bos taurus GN=TAGLN2 PE=2 SV=3	Q5E9F5	22583.23	0.04%
37	>sp|Q9N2I2|IPSP_BOVIN Plasma serine protease inhibitor OS=Bos taurus GN=SERPINA5 PE=1 SV=1	Q9N2I2	45439.06	0.04%
38	>sp|Q5E9B1|LDHB_BOVIN L-lactate dehydrogenase B chain OS=Bos taurus GN=LDHB PE=2 SV=4	Q5E9B1	36985.35	0.04%

39	>sp|P81644|APOA2_BOVIN Apolipoprotein A-II OS=Bos taurus GN=APOA2 PE=1 SV=2	P81644	11251.96	0.04%
40	>tr|G5E5T5|G5E5T5_BOVIN Uncharacterized protein OS=Bos taurus PE=1 SV=1	G5E5T5	44457.32	0.03%
41	>sp|P19120|HSP7C_BOVIN Heat shock cognate 71 kDa protein OS=Bos taurus GN=HSPA8 PE=1 SV=2	P19120	71424.52	0.02%
42	>tr|F1MQ37|F1MQ37_BOVIN Uncharacterized protein OS=Bos taurus GN=MYH9 PE=1 SV=2	F1MQ37	228215.14	0.02%
43	>sp|Q27975|HS71A_BOVIN Heat shock 70 kDa protein 1A OS=Bos taurus GN=HSPA1A PE=1 SV=2	Q27975	70500.34	0.02%
44	>tr|A5D7S8|A5D7S8_BOVIN Fibulin-1 OS=Bos taurus GN=FBLN1 PE=2 SV=1	A5D7S8	81526.98	0.02%
45	>tr|F1MW44|F1MW44_BOVIN Coagulation factor XIII A chain OS=Bos taurus GN=F13A1 PE=4 SV=1	F1MW44	83192.81	0.02%
46	>sp|Q3SZ54|IF4A1_BOVIN Eukaryotic initiation factor 4A-I OS=Bos taurus GN=EIF4A1 PE=2 SV=1	Q3SZ54	46352.67	0.02%
47	>tr|Q3SZH5|Q3SZH5_BOVIN Angiotensinogen OS=Bos taurus GN=AGT PE=1 SV=1	Q3SZH5	45712.65	0.01%
48	>tr|A5D7L1|A5D7L1_BOVIN CLEC11A protein OS=Bos taurus GN=CLEC11A PE=2 SV=1	A5D7L1	35934.69	0.01%
49	>sp|P00978|AMBP_BOVIN Protein AMBP OS=Bos taurus GN=AMBP PE=1 SV=2	P00978	40121.69	0.01%
50	>sp|Q76LV2|HS90A_BOVIN Heat shock protein HSP 90-alpha OS=Bos taurus GN=HSP90AA1 PE=1 SV=3	Q76LV2	85076.86	0.01%
51	>tr|Q58DL9|Q58DL9_BOVIN Phospholipid transfer protein OS=Bos taurus GN=PLTP PE=2 SV=1	Q58DL9	56620.7	0.01%
52	>sp|Q3SX14|GELS_BOVIN Gelsolin OS=Bos taurus GN=GSN PE=2 SV=1	Q3SX14	80965.66	0.01%
53	>sp|Q2HJB8|TBA8_BOVIN Tubulin alpha-8 chain OS=Bos taurus GN=TUBA8 PE=2 SV=1	Q2HJB8	50705.82	0.01%
54	>sp|Q29443|TRFE_BOVIN Serotransferrin OS=Bos taurus GN=TF PE=2 SV=1	Q29443	79869.55	0.01%
55	>tr|E1BJK2|E1BJK2_BOVIN Tubulin beta chain OS=Bos taurus GN=TUBB1 PE=3 SV=1	E1BJK2	50582.75	0.01%
56	>tr|F1MNW4|F1MNW4_BOVIN Inter-alpha-trypsin inhibitor heavy chain H2 OS=Bos taurus GN=ITIH2 PE=1 SV=2	F1MNW4	106546.01	0.00%
57	>tr|F1N102|F1N102_BOVIN Uncharacterized protein OS=Bos taurus GN=C8B PE=4 SV=2	F1N102	68268.09	0.00%
58	>sp|Q28065|C4BPA_BOVIN C4b-binding protein alpha chain OS=Bos taurus GN=C4BPA PE=2 SV=1	Q28065	70951.3	0.00%
**NP03**				
1	>sp|P02081|HBBF_BOVIN Hemoglobin fetal subunit	P02081	15963.34	35.85%

	beta OS=Bos taurus PE=1 SV=1			
2	>tr|V6F9A2|V6F9A2_BOVIN Apolipoprotein A-I preproprotein OS=Bos taurus GN=APOA1 PE=3 SV=1	V6F9A2	30257.9	12.01%
3	>sp|P41361|ANT3_BOVIN Antithrombin-III OS=Bos taurus GN=SERPINC1 PE=1 SV=2	P41361	52827.16	8.08%
4	>tr|A0A140T881|A0A140T881_BOVIN Apolipoprotein E OS=Bos taurus GN=APOE PE=3 SV=1	A0A140T881	36073.61	5.74%
5	>sp|P02769|ALBU_BOVIN Serum albumin OS=Bos taurus GN=ALB PE=1 SV=4	P02769	71244.31	4.79%
6	>sp|P00735|THRB_BOVIN Prothrombin OS=Bos taurus GN=F2 PE=1 SV=2	P00735	71886.34	4.24%
7	>tr|Q3ZBS7|Q3ZBS7_BOVIN Vitronectin OS=Bos taurus GN=VTN PE=1 SV=1	Q3ZBS7	54339.32	3.86%
8	>sp|P01966|HBA_BOVIN Hemoglobin subunit alpha OS=Bos taurus GN=HBA PE=1 SV=2	P01966	15174.93	3.78%
9	>sp|P02070|HBB_BOVIN Hemoglobin subunit beta OS=Bos taurus GN=HBB PE=1 SV=1	P02070	16001.32	2.21%
10	>tr|F1MDH3|F1MDH3_BOVIN Talin 1 OS=Bos taurus GN=TLN1 PE=1 SV=2	F1MDH3	272810.53	2.16%
11	>sp|P61223|RAP1B_BOVIN Ras-related protein Rap-1b OS=Bos taurus GN=RAP1B PE=2 SV=1	P61223	21039.67	1.66%
12	>tr|A6QPP2|A6QPP2_BOVIN SERPIND1 protein OS=Bos taurus GN=SERPIND1 PE=2 SV=1	A6QPP2	55457.54	1.65%
13	>sp|Q2UVX4|CO3_BOVIN Complement C3 OS=Bos taurus GN=C3 PE=1 SV=2	Q2UVX4	188674	1.44%
14	>tr|G3N0S9|G3N0S9_BOVIN Uncharacterized protein OS=Bos taurus GN=LOC515150 PE=4 SV=1	G3N0S9	22948.58	1.26%
15	>sp|P84080|ARF1_BOVIN ADP-ribosylation factor 1 OS=Bos taurus GN=ARF1 PE=1 SV=2	P84080	20740.68	1.26%
16	>sp|P52556|BLVRB_BOVIN Flavin reductase (NADPH) OS=Bos taurus GN=BLVRB PE=1 SV=2	P52556	22232.4	1.14%
17	>sp|Q32KY0|APOD_BOVIN Apolipoprotein D OS=Bos taurus GN=APOD PE=2 SV=1	Q32KY0	21615.79	0.91%
18	>sp|Q7SIH1|A2MG_BOVIN Alpha-2-macroglobulin OS=Bos taurus GN=A2M PE=1 SV=2	Q7SIH1	168952.25	0.85%
19	>sp|P34955|A1AT_BOVIN Alpha-1-antiproteinase OS=Bos taurus GN=SERPINA1 PE=1 SV=1	P34955	46417.03	0.70%
20	>sp|P17697|CLUS_BOVIN Clusterin OS=Bos taurus GN=CLU PE=1 SV=1	P17697	51651.37	0.48%
21	>sp|P60712|ACTB_BOVIN Actin, cytoplasmic 1 OS=Bos taurus GN=ACTB PE=1 SV=1	P60712	42051.85	0.40%
22	>sp|P81644|APOA2_BOVIN Apolipoprotein A-II OS=Bos taurus GN=APOA2 PE=1 SV=2	P81644	11251.96	0.38%

23	>sp|Q9N2I2|IPSP_BOVIN Plasma serine protease inhibitor OS=Bos taurus GN=SERPINA5 PE=1 SV=1	Q9N2I2	45439.06	0.35%
24	>tr|A5D7E0|A5D7E0_BOVIN RAB35 protein OS=Bos taurus GN=RAB35 PE=2 SV=1	A5D7E0	23295.88	0.32%
25	>sp|Q2HJI8|RAB8B_BOVIN Ras-related protein Rab-8B OS=Bos taurus GN=RAB8B PE=2 SV=1	Q2HJI8	23812.17	0.31%
26	>sp|P10096|G3P_BOVIN Glyceraldehyde-3-phosphate dehydrogenase OS=Bos taurus GN=GAPDH PE=1 SV=4	P10096	36073.38	0.27%
27	>sp|Q3SZF2|ARF4_BOVIN ADP-ribosylation factor 4 OS=Bos taurus GN=ARF4 PE=2 SV=3	Q3SZF2	20628.64	0.27%
28	>tr|A5D7S8|A5D7S8_BOVIN Fibulin-1 OS=Bos taurus GN=FBLN1 PE=2 SV=1	A5D7S8	81526.98	0.26%
29	>tr|F1MND1|F1MND1_BOVIN Cell division control protein 42 homolog OS=Bos taurus GN=CDC42 PE=1 SV=1	F1MND1	21142.14	0.26%
30	>sp|Q28178|TSP1_BOVIN Thrombospondin-1 OS=Bos taurus GN=THBS1 PE=2 SV=2	Q28178	133441.97	0.24%
31	>sp|Q5E9B1|LDHB_BOVIN L-lactate dehydrogenase B chain OS=Bos taurus GN=LDHB PE=2 SV=4	Q5E9B1	36985.35	0.22%
32	>sp|Q3SX14|GELS_BOVIN Gelsolin OS=Bos taurus GN=GSN PE=2 SV=1	Q3SX14	80965.66	0.21%
33	>sp|P81948|TBA4A_BOVIN Tubulin alpha-4A chain OS=Bos taurus GN=TUBA4A PE=1 SV=2	P81948	50633.66	0.20%
34	>sp|Q5E9F5|TAGL2_BOVIN Transgelin-2 OS=Bos taurus GN=TAGLN2 PE=2 SV=3	Q5E9F5	22583.23	0.19%
35	>sp|P62739|ACTA_BOVIN Actin, aortic smooth muscle OS=Bos taurus GN=ACTA2 PE=1 SV=1	P62739	42380.95	0.16%
36	>sp|Q28107|FA5_BOVIN Coagulation factor V OS=Bos taurus GN=F5 PE=1 SV=1	Q28107	249909.67	0.14%
37	>sp|P80109|PHLD_BOVIN Phosphatidylinositol-glycan-specific phospholipase D OS=Bos taurus GN=GPLD1 PE=1 SV=1	P80109	93170.32	0.13%
38	>tr|G5E513|G5E513_BOVIN Uncharacterized protein OS=Bos taurus PE=1 SV=1	G5E513	50622.92	0.12%
39	>sp|Q3T0P6|PGK1_BOVIN Phosphoglycerate kinase 1 OS=Bos taurus GN=PGK1 PE=2 SV=3	Q3T0P6	44908.24	0.11%
40	>tr|Q3SZH5|Q3SZH5_BOVIN Angiotensinogen OS=Bos taurus GN=AGT PE=1 SV=1	Q3SZH5	45712.65	0.11%
41	>sp|A6QLY7|PBIP1_BOVIN Pre-B-cell leukemia transcription factor-interacting protein 1 OS=Bos taurus GN=PBXIP1 PE=2 SV=1	A6QLY7	80551.16	0.10%
42	>tr|A5D984|A5D984_BOVIN Pyruvate kinase OS=Bos taurus GN=PKM2 PE=1 SV=1	A5D984	58482.34	0.09%

43	>sp|P00978|AMBP_BOVIN Protein AMBP OS=Bos taurus GN=AMBP PE=1 SV=2	P00978	40121.69	0.08%
44	>tr|F1MZC0|F1MZC0_BOVIN Uncharacterized protein OS=Bos taurus PE=1 SV=1	F1MZC0	30425.02	0.08%
45	>sp|P19858|LDHA_BOVIN L-lactate dehydrogenase A chain OS=Bos taurus GN=LDHA PE=2 SV=2	P19858	36916.39	0.07%
46	>sp|Q28065|C4BPA_BOVIN C4b-binding protein alpha chain OS=Bos taurus GN=C4BPA PE=2 SV=1	Q28065	70951.3	0.06%
47	>sp|Q6B856|TBB2B_BOVIN Tubulin beta-2B chain OS=Bos taurus GN=TUBB2B PE=1 SV=2	Q6B856	50377.13	0.06%
48	>tr|E1BNR0|E1BNR0_BOVIN Uncharacterized protein OS=Bos taurus GN=APOB PE=1 SV=2	E1BNR0	516807	0.05%
49	>sp|Q3SYV4|CAP1_BOVIN Adenylyl cyclase-associated protein 1 OS=Bos taurus GN=CAP1 PE=2 SV=3	Q3SYV4	51582.59	0.05%
50	>sp|Q3MHL4|SAHH_BOVIN Adenosylhomocysteinase OS=Bos taurus GN=AHCY PE=2 SV=3	Q3MHL4	48120.49	0.05%
51	>tr|F1MFW9|F1MFW9_BOVIN Keratin 24 OS=Bos taurus GN=KRT24 PE=3 SV=2	F1MFW9	50903.66	0.04%
52	>sp|Q3T056|LDH6B_BOVIN L-lactate dehydrogenase A-like 6B OS=Bos taurus GN=LDHAL6B PE=2 SV=1	Q3T056	42022.01	0.04%
53	>sp|Q32LP0|URP2_BOVIN Fermitin family homolog 3 OS=Bos taurus GN=FERMT3 PE=2 SV=1	Q32LP0	76305.05	0.04%
54	>tr|G5E5T5|G5E5T5_BOVIN Uncharacterized protein OS=Bos taurus PE=1 SV=1	G5E5T5	44457.32	0.04%
55	>tr|Q2KJC7|Q2KJC7_BOVIN Periostin variant 7 OS=Bos taurus GN=POSTN PE=1 SV=1	Q2KJC7	87488.8	0.04%
56	>tr|Q0II79|Q0II79_BOVIN TLN1 protein (Fragment) OS=Bos taurus GN=TLN1 PE=2 SV=1	Q0II79	47570.7	0.03%
57	>sp|Q08E20|ESTD_BOVIN S-formylglutathione hydrolase OS=Bos taurus GN=ESD PE=2 SV=1	Q08E20	32040.72	0.03%
58	>sp|Q3SZB7|F16P1_BOVIN Fructose-1,6-bisphosphatase 1 OS=Bos taurus GN=FBP1 PE=2 SV=3	Q3SZB7	37047.02	0.03%
59	>sp|P28800|A2AP_BOVIN Alpha-2-antiplasmin OS=Bos taurus GN=SERPINF2 PE=1 SV=2	P28800	54961.29	0.03%
60	>tr|Q58DL9|Q58DL9_BOVIN Phospholipid transfer protein OS=Bos taurus GN=PLTP PE=2 SV=1	Q58DL9	56620.7	0.03%
61	>tr|Q2TBQ1|Q2TBQ1_BOVIN Coagulation factor XIII, B polypeptide OS=Bos taurus GN=F13B PE=2 SV=1	Q2TBQ1	77398.69	0.02%
62	>tr|A5D7E1|A5D7E1_BOVIN MGC139254 protein OS=Bos taurus GN=MGC139254 PE=2 SV=1	A5D7E1	60949.8	0.02%
63	>sp|Q9XSJ4|ENOA_BOVIN Alpha-enolase OS=Bos taurus GN=ENO1 PE=1 SV=4	Q9XSJ4	47638.55	0.02%

64	>sp|Q3MHN5|VTDB_BOVIN Vitamin D-binding protein OS=Bos taurus GN=GC PE=2 SV=1	Q3MHN5	54903.71	0.02%
65	>tr|F6QTA8|F6QTA8_BOVIN Uncharacterized protein OS=Bos taurus GN=PARVA PE=4 SV=1	F6QTA8	39233.89	0.02%
66	>tr|Q1RMN9|Q1RMN9_BOVIN C4b-binding protein alpha-like OS=Bos taurus GN=LOC510860 PE=2 SV=1	Q1RMN9	22723.4	0.02%
67	>sp|Q0VCU1|ACOC_BOVIN Cytoplasmic aconitate hydratase OS=Bos taurus GN=ACO1 PE=2 SV=1	Q0VCU1	98711.97	0.02%
68	>sp|Q2HJF4|PBLD_BOVIN Phenazine biosynthesis-like domain-containing protein OS=Bos taurus GN=PBLD PE=2 SV=1	Q2HJF4	32091.54	0.01%
69	>sp|P19217|ST1E1_BOVIN Estrogen sulfotransferase OS=Bos taurus GN=SULT1E1 PE=1 SV=1	P19217	34845.42	0.01%
70	>sp|Q58D31|DHSO_BOVIN Sorbitol dehydrogenase OS=Bos taurus GN=SORD PE=2 SV=3	Q58D31	38644.91	0.01%
71	>tr|F1MS85|F1MS85_BOVIN Uncharacterized protein OS=Bos taurus PE=4 SV=1	F1MS85	50985.84	0.01%
72	>tr|F1MJK3|F1MJK3_BOVIN Uncharacterized protein OS=Bos taurus PE=4 SV=2	F1MJK3	166988.41	0.01%
73	>sp|Q3SZP2|MARE2_BOVIN Microtubule-associated protein RP/EB family member 2 OS=Bos taurus GN=MAPRE2 PE=2 SV=1	Q3SZP2	37193.25	0.01%
74	>sp|P19483|ATPA_BOVIN ATP synthase subunit alpha, mitochondrial OS=Bos taurus GN=ATP5A1 PE=1 SV=1	P19483	59796.83	0.01%
75	>sp|Q2KJ83|CBPN_BOVIN Carboxypeptidase N catalytic chain OS=Bos taurus GN=CPN1 PE=2 SV=1	Q2KJ83	52920.8	0.01%
76	>sp|Q2KJC6|METK1_BOVIN S-adenosylmethionine synthase isoform type-1 OS=Bos taurus GN=MAT1A PE=2 SV=1	Q2KJC6	44303.13	0.01%
77	>tr|F1N169|F1N169_BOVIN Uncharacterized protein OS=Bos taurus GN=FLNA PE=1 SV=2	F1N169	285282.59	0.01%
78	>tr|F1MW44|F1MW44_BOVIN Coagulation factor XIII A chain OS=Bos taurus GN=F13A1 PE=4 SV=1	F1MW44	83192.81	0.00%
79	>tr|M5FKF4|M5FKF4_BOVIN Insulin-like growth factor binding protein, acid labile subunit OS=Bos taurus GN=IGFALS PE=4 SV=1	M5FKF4	66636.46	0.00%
80	>sp|Q0VCK0|PUR9_BOVIN Bifunctional purine biosynthesis protein PURH OS=Bos taurus GN=ATIC PE=2 SV=1	Q0VCK0	65012.51	0.00%
81	>tr|F1MVK1|F1MVK1_BOVIN Uncharacterized protein OS=Bos taurus PE=4 SV=2	F1MVK1	175175.17	0.00%
82	>tr|F1MQ37|F1MQ37_BOVIN Uncharacterized protein OS=Bos taurus GN=MYH9 PE=1 SV=2	F1MQ37	228215.14	0.00%

<table>
<tr><td colspan="5">NP04</td></tr>
<tr><td>1</td><td>>sp|P02081|HBBF_BOVIN Hemoglobin fetal subunit beta OS=Bos taurus PE=1 SV=1</td><td>P02081</td><td>15963.34</td><td>26.50%</td></tr>
<tr><td>2</td><td>>sp|P02769|ALBU_BOVIN Serum albumin OS=Bos taurus GN=ALB PE=1 SV=4</td><td>P02769</td><td>71244.31</td><td>23.09%</td></tr>
<tr><td>3</td><td>>tr|V6F9A2|V6F9A2_BOVIN Apolipoprotein A-I preproprotein OS=Bos taurus GN=APOA1 PE=3 SV=1</td><td>V6F9A2</td><td>30257.9</td><td>18.63%</td></tr>
<tr><td>4</td><td>>sp|P01966|HBA_BOVIN Hemoglobin subunit alpha OS=Bos taurus GN=HBA PE=1 SV=2</td><td>P01966</td><td>15174.93</td><td>17.79%</td></tr>
<tr><td>5</td><td>>tr|A0A140T881|A0A140T881_BOVIN Apolipoprotein E OS=Bos taurus GN=APOE PE=3 SV=1</td><td>A0A140T881</td><td>36073.61</td><td>5.30%</td></tr>
<tr><td>6</td><td>>sp|P12763|FETUA_BOVIN Alpha-2-HS-glycoprotein OS=Bos taurus GN=AHSG PE=1 SV=2</td><td>P12763</td><td>39192.69</td><td>1.64%</td></tr>
<tr><td>7</td><td>>tr|Q3ZBS7|Q3ZBS7_BOVIN Vitronectin OS=Bos taurus GN=VTN PE=1 SV=1</td><td>Q3ZBS7</td><td>54339.32</td><td>1.11%</td></tr>
<tr><td>8</td><td>>sp|P34955|A1AT_BOVIN Alpha-1-antiproteinase OS=Bos taurus GN=SERPINA1 PE=1 SV=1</td><td>P34955</td><td>46417.03</td><td>1.09%</td></tr>
<tr><td>9</td><td>>tr|F1N1I6|F1N1I6_BOVIN Gelsolin OS=Bos taurus GN=GSN PE=1 SV=1</td><td>F1N1I6</td><td>85976.38</td><td>0.69%</td></tr>
<tr><td>10</td><td>>sp|P41361|ANT3_BOVIN Antithrombin-III OS=Bos taurus GN=SERPINC1 PE=1 SV=2</td><td>P41361</td><td>52827.16</td><td>0.55%</td></tr>
<tr><td>11</td><td>>sp|P81644|APOA2_BOVIN Apolipoprotein A-II OS=Bos taurus GN=APOA2 PE=1 SV=2</td><td>P81644</td><td>11251.96</td><td>0.41%</td></tr>
<tr><td>12</td><td>>tr|Q3SZH5|Q3SZH5_BOVIN Angiotensinogen OS=Bos taurus GN=AGT PE=1 SV=1</td><td>Q3SZH5</td><td>45712.65</td><td>0.36%</td></tr>
<tr><td>13</td><td>>sp|P68103|EF1A1_BOVIN Elongation factor 1-alpha 1 OS=Bos taurus GN=EEF1A1 PE=1 SV=1</td><td>P68103</td><td>50451.24</td><td>0.32%</td></tr>
<tr><td>14</td><td>>sp|P60712|ACTB_BOVIN Actin, cytoplasmic 1 OS=Bos taurus GN=ACTB PE=1 SV=1</td><td>P60712</td><td>42051.85</td><td>0.26%</td></tr>
<tr><td>15</td><td>>sp|P61223|RAP1B_BOVIN Ras-related protein Rap-1b OS=Bos taurus GN=RAP1B PE=2 SV=1</td><td>P61223</td><td>21039.67</td><td>0.25%</td></tr>
<tr><td>16</td><td>>sp|P84080|ARF1_BOVIN ADP-ribosylation factor 1 OS=Bos taurus GN=ARF1 PE=1 SV=2</td><td>P84080</td><td>20740.68</td><td>0.25%</td></tr>
<tr><td>17</td><td>>sp|P00735|THRB_BOVIN Prothrombin OS=Bos taurus GN=F2 PE=1 SV=2</td><td>P00735</td><td>71886.34</td><td>0.21%</td></tr>
<tr><td>18</td><td>>sp|Q5E9F5|TAGL2_BOVIN Transgelin-2 OS=Bos taurus GN=TAGLN2 PE=2 SV=3</td><td>Q5E9F5</td><td>22583.23</td><td>0.18%</td></tr>
<tr><td>19</td><td>>sp|Q3MHN5|VTDB_BOVIN Vitamin D-binding protein OS=Bos taurus GN=GC PE=2 SV=1</td><td>Q3MHN5</td><td>54903.71</td><td>0.18%</td></tr>
<tr><td>20</td><td>>tr|E1B6Z6|E1B6Z6_BOVIN Uncharacterized protein OS=Bos taurus GN=LCN2 PE=1 SV=2</td><td>E1B6Z6</td><td>23082.95</td><td>0.17%</td></tr>
<tr><td>21</td><td>>sp|P10096|G3P_BOVIN Glyceraldehyde-3-phosphate dehydrogenase OS=Bos taurus GN=GAPDH PE=1</td><td>P10096</td><td>36073.38</td><td>0.16%</td></tr>
</table>

<table>
<tr><td></td><td>SV=4</td><td></td><td></td><td></td></tr>
<tr><td>22</td><td>>sp|Q3T0P6|PGK1_BOVIN Phosphoglycerate kinase 1 OS=Bos taurus GN=PGK1 PE=2 SV=3</td><td>Q3T0P6</td><td>44908.24</td><td>0.11%</td></tr>
<tr><td>23</td><td>>sp|Q2UVX4|CO3_BOVIN Complement C3 OS=Bos taurus GN=C3 PE=1 SV=2</td><td>Q2UVX4</td><td>188674</td><td>0.09%</td></tr>
<tr><td>24</td><td>>tr|G5E607|G5E607_BOVIN Uncharacterized protein OS=Bos taurus PE=4 SV=1</td><td>G5E607</td><td>62521.15</td><td>0.09%</td></tr>
<tr><td>25</td><td>>sp|Q28107|FA5_BOVIN Coagulation factor V OS=Bos taurus GN=F5 PE=1 SV=1</td><td>Q28107</td><td>249909.67</td><td>0.08%</td></tr>
<tr><td>26</td><td>>sp|P17697|CLUS_BOVIN Clusterin OS=Bos taurus GN=CLU PE=1 SV=1</td><td>P17697</td><td>51651.37</td><td>0.08%</td></tr>
<tr><td>27</td><td>>tr|G3N3N1|G3N3N1_BOVIN Uncharacterized protein OS=Bos taurus GN=ARF6 PE=3 SV=1</td><td>G3N3N1</td><td>20183.47</td><td>0.07%</td></tr>
<tr><td>28</td><td>>tr|A6QPP2|A6QPP2_BOVIN SERPIND1 protein OS=Bos taurus GN=SERPIND1 PE=2 SV=1</td><td>A6QPP2</td><td>55457.54</td><td>0.07%</td></tr>
<tr><td>29</td><td>>sp|Q9N2I2|IPSP_BOVIN Plasma serine protease inhibitor OS=Bos taurus GN=SERPINA5 PE=1 SV=1</td><td>Q9N2I2</td><td>45439.06</td><td>0.06%</td></tr>
<tr><td>30</td><td>>sp|Q5E9B1|LDHB_BOVIN L-lactate dehydrogenase B chain OS=Bos taurus GN=LDHB PE=2 SV=4</td><td>Q5E9B1</td><td>36985.35</td><td>0.04%</td></tr>
<tr><td>31</td><td>>sp|Q58D62|FETUB_BOVIN Fetuin-B OS=Bos taurus GN=FETUB PE=1 SV=1</td><td>Q58D62</td><td>43434.41</td><td>0.03%</td></tr>
<tr><td>32</td><td>>tr|F2Z4H6|F2Z4H6_BOVIN Uncharacterized protein OS=Bos taurus GN=TRIM23 PE=4 SV=1</td><td>F2Z4H6</td><td>66319.23</td><td>0.02%</td></tr>
<tr><td>33</td><td>>tr|F1MFW9|F1MFW9_BOVIN Keratin 24 OS=Bos taurus GN=KRT24 PE=3 SV=2</td><td>F1MFW9</td><td>50903.66</td><td>0.02%</td></tr>
<tr><td>34</td><td>>sp|Q3T052|ITIH4_BOVIN Inter-alpha-trypsin inhibitor heavy chain H4 OS=Bos taurus GN=ITIH4 PE=1 SV=1</td><td>Q3T052</td><td>101620.61</td><td>0.02%</td></tr>
<tr><td>35</td><td>>sp|P19879|MIME_BOVIN Mimecan OS=Bos taurus GN=OGN PE=1 SV=2</td><td>P19879</td><td>34530</td><td>0.02%</td></tr>
<tr><td>36</td><td>>sp|Q95121|PEDF_BOVIN Pigment epithelium-derived factor OS=Bos taurus GN=SERPINF1 PE=1 SV=1</td><td>Q95121</td><td>46314.35</td><td>0.01%</td></tr>
<tr><td>37</td><td>>sp|Q3SZB7|F16P1_BOVIN Fructose-1,6-bisphosphatase 1 OS=Bos taurus GN=FBP1 PE=2 SV=3</td><td>Q3SZB7</td><td>37047.02</td><td>0.01%</td></tr>
<tr><td>38</td><td>>tr|A7MBA2|A7MBA2_BOVIN PSMD1 protein OS=Bos taurus GN=PSMD1 PE=1 SV=1</td><td>A7MBA2</td><td>106845.2</td><td>0.01%</td></tr>
<tr><td>39</td><td>>sp|Q27975|HS71A_BOVIN Heat shock 70 kDa protein 1A OS=Bos taurus GN=HSPA1A PE=1 SV=2</td><td>Q27975</td><td>70500.34</td><td>0.01%</td></tr>
<tr><td>40</td><td>>sp|Q28178|TSP1_BOVIN Thrombospondin-1 OS=Bos taurus GN=THBS1 PE=2 SV=2</td><td>Q28178</td><td>133441.97</td><td>0.01%</td></tr>
<tr><td>41</td><td>>tr|F1MDH3|F1MDH3_BOVIN Talin 1 OS=Bos taurus GN=TLN1 PE=1 SV=2</td><td>F1MDH3</td><td>272810.53</td><td>0.00%</td></tr>
</table>
